# Controlled comparison evaluation of the soothing effect of 3 cosmetic products on skin discomfort induced by an irritant chemical agent (capsaicin)

**DOI:** 10.1111/jocd.16526

**Published:** 2024-09-05

**Authors:** Maria Vitale, Ana López, Maria Teresa Truchuelo, Vincenzo Nobile, Massimo Milani, María José Gómez‐Sánchez

**Affiliations:** ^1^ Medical Affairs Department Cantabria Labs Madrid Spain; ^2^ R&D Department Cantabria Labs Madrid Spain; ^3^ Vithas Nuestra Señora de América Hospital Madrid Spain; ^4^ R&D Department Complife Italia S.r.l. Pavia Italy; ^5^ Medical Department Cantabria Labs Difa Cooper Varese Italy; ^6^ Medical affairs Department Cantabria Labs Madrid Spain

**Keywords:** Aquammunist, burning, sensitive skin, thermal spring water, TRPV1

## Abstract

**Background:**

Sensitive skin is a highly prevalent problem. The objective of the study was to assess whether the tested products are effective and safe in terms of improving the symptoms of sensitive skin.

**Methods:**

A clinical randomized split‐face study was carried out on 24 healthy female subjects. Three cosmetic combinations were tested versus vehicle: product A (Solía Thermal Spring Water–TSW–from Cantabria, Spain + diatom algae–*P. tricornutum*–extract), product B (Solía TSW + diatom algae extract + *Annona cherimola* Fruit Extract) and product C (Solía TSW + diatom algae extract + *Annona cherimola* Fruit Extract + niacinamide). Prior to each application of the study Product (A, B, or C)/vehicle, 10% of aqueous solution of capsaicin to induce skin irritation was applied, mimicking the symptoms of sensitive skin. Stinging and burning sensations were evaluated at different time points.

**Results:**

All three tested products A, B, and C showed to act better in calming the symptoms induced by capsaicin when compared to the vehicle.

**Conclusions:**

The tested products would be an interesting option for treating stinging and burning sensations in sensitive skin patients.

## 1 BACKGROUND

Sensitive skin (SS), also named as reactive, hyper‐reactive, intolerant, or irritable skin, is defined as the onset of erythema and/or unpleasant sensations (stinging, burning, prickling, tingling, pain, pruritus) in response to stimuli that should not provoke them, without skin lesions that would explain them and in the absence of underlying skin lesions.^1^ SS must be distinguished from irritated skin and sensitized skin.^2^ The subjective symptoms and lack of visible specific manifestations, make the diagnosis of SS challenging. Some predisposing factors include extrinsic factors such as physical (pollution, ultraviolet radiation, heat, cold, wind…), or chemical factors (cosmetics, soap, water, and pollution), and intrinsic factors such as psychological (stress) or increased hormonal presence in women.^1^, ^3^ A recent meta‐analysis showed that the most important triggering factor declared by subjects is the use of cosmetics.^4^

According to Berardesca et al, the prevalence of self‐declared SS has been reported to affect up to 70% of the population.^5^ The pathophysiology of SS has not been completely well established. A multifactorial origin has been proposed. One feature of SS is a decrease in “skin's resistance,” not directly related to any immunological or allergic mechanism; and another feature, probably the main one, it would be a sensory dysfunction and a barrier skin function alteration. (Table 1) Furthermore, there is a recent publication that showed a relationship between anxiety and sensitive skin. The HADS‐Anxiety scores were found to be significantly higher in patients with sensitive skin and a strong positive correlation was found between the HADS‐Anxiety scores and the erythema index in these patients.^20^

**TABLE 1:** Multifactorial origin of SS.

*Anatomical changes* 3, 6	Functional changes: Changes in physico‐chemical properties, immunology, and microflora impact the strength of the skin^7^, ^8^, ^9^, ^10^			
Differences in thickness, hydration, and innervations of the stratum corneum and/or epidermis^3^, ^11^	Chemical: Natural moisturizing factor, lipids, human defensins, cathelicidins, pH	Physical: Corneocytes, lipids, keratinocyte, tight junctions	Immunological: Antigen presenting cells, innate lymphoid cells, memory cells	Microbiome: Commensal and other microorganisms
*Vascular hyperreactivity*: Even present without erythema or visible inflammation signs^12^	*Neurosensory dysfunction*: Is one of the main pathological mechanisms explaining the sensory symptoms^13^, ^14^			
Significant difference in vascular depth, shape, and density between SS and normal skin.^12^ Thus, testing vasodilation may be an objective approach to study sensitive skin.^1^	Cutaneous nerves: Alteration of neurosensory activity, inadequate intra‐epidermal nerve fiber protection, altered nerve endings, decreased intra‐epidermal nerve fiber density (mainly peptidergic C‐fibers),^14^ increased neurotransmitter release,^15^ slower neurotransmitter removal^16^	Hyper‐activation of proteins and receptors: Hyperactivation of proteins that allow the perception of multiple environmental factors on the surface of keratinocytes and intra‐epidermal nerve endings^17^	Up‐regulation of TRPV1: TRPV1 is known to mediate skin reactivity, flushing, burning, itching, and stinging sensations when stimulated.^18^ TRPV1 expression is upregulated in subjects with SS, and it correlates with the intensity of the symptoms.^19^ Keratinocytes express TRPV1/TRPV4 which is coupled to pruritus receptors, possibly to amplify and optimize sensory perception^17^	

As for the classification of SS, the special interest group on SS of the International Forum for the Study of Itch (IFSI), referred to three types based on their physiological parameters: type I (low barrier function group), type II (inflammation group with normal barrier function and inflammatory changes) and type III (pseudo‐ healthy group in terms of normal barrier function and no inflammatory changes).^1^

Going deeper into the diagnosis of SS, it is possible to use the reactions to TRPs agonists as an indicator of SS.^21^ As capsaicin is a natural activator of TRPV1 and it is known to correlate with sensitive skin, the capsaicin assay is a standard experimental setting, used to investigate skin calming effects of cosmetic products,^22^, ^23^ this is why this method was selected for the present study. There is no simple treatment or ingredient for managing sensitive skin. It involves dedicated research to find the combination of different ingredients to provide an efficient treatment strategy. The purpose of this work was to show the efficacy of different active natural ingredients or extract combinations (that had previously shown to have benefits in different alterations observed in the pathogenesis of SS), to induce calming effects on the skin after capsaicin‐induced irritation.

The first one of the tested cosmetic combinations called Aquammunist® by Cantabrialabs, is a mix of Solía Thermal Spring Water (Solía TSW), a mesothermal water from a hot spring located in the north of Spain (Villaescusa, Cantabria), plus a diatom algae (*Phaeodactylum tricornutum*) extract. On one hand, thermal water is commonly used in SS products due to its soothing, moisturizing, and anti‐inflammatory properties.^24^, ^25^, ^26^ On the other hand, *P. tricornutum* is a marine diatom algae with documented benefits in sensitive skin treatments.^27^ Diatoms are commonly used in the pharmaceutical^28^ and cosmetic industries.^29^ In particular, *P. tricornutum* extracts are used for their anti‐inflammatory and barrier function repair activities,^30^, ^31^ being fucoxanthin the bioactive molecule bearing the most relevant anti‐inflammatory properties by controlling the levels of pro‐inflammatory cytokines such as IL‐1β, IL‐6, and TNF‐α.^30^ Specifically, the diatom extract used in this work consists of *P. tricornutum* extract encapsulated in liposomes, containing omega‐3 fatty acids, and standardized to fucoxanthin levels. Having in mind the individual properties described above and comprehensive preclinical data (manuscript under submission), this work clinically tested for the first time the combination of these two components in the form of a single ingredient, Aquammunist®.

The second treatment consists of a combination of Aquammunist®, plus *Annona Cherimola* Fruit Extract. *Annona* genus of plants has been reported to contain different bioactive molecules stimulating the skin's endogenous adaptive mechanisms which is able to palliate the hyperactivation of TRPV1 receptors.^31^, ^32^, ^33^ This general evidence has been supported by a comprehensive preliminary characterization of the ingredient (data on file).^34^ Thus, considering the characteristic neurosensory dysfunctions found in SS individuals, and the mentioned correlation of TRPV upregulation with the symptoms, we evaluated the contribution of *A. Cherimola* Fruit Extract in the mentioned capsaicin model to investigate skin calming effects.

Finally, the third treatment combined the previously described second treatment with niacinamide, a well‐known antioxidant, with anti‐inflammatory and barrier function strengthening properties.^35^

## 2 OBJECTIVES

The study aimed to assess the efficacy of 3 cosmetic combinations in decreasing the skin discomforts induced by capsaicin. The ingredients used in this study had previously reported beneficial properties for irritated skin.^24^, ^25^, ^26^, ^27^, ^28^, ^29^, ^30^

## 3 MATERIALS AND METHODS

### 3.1 Study design

This was a randomized, clinical split‐face study. The study met all ethical requirements. All the subjects were informed about the test procedures and signed a consent form.

### 3.2 Study participants

24 healthy female subjects, aged over 18 years old (27–65 years old) were enrolled. The inclusion criteria were as followed: healthy female aged over 18 years old, have positive reaction to stinging test with capsaicin, with pharmacological therapy stable for at least 1 month without any changes expected or planned during the study, commitment not to change the daily routine or the lifestyle, commitment not to use during the study period other products with the same effect of the tested product, absence of previous allergy for topical products. All subjects understood the information about the test procedures, participated in the study voluntarily, and signed the informed consent form. The exclusion criteria were as follows: pregnant or lactating women; with any acute or chronic diseases or skin disorders able to interfere with the outcome of the study, subjects participating or planning to participate in other clinical trials, subjects under pharmacological treatment incompatible with the requirements of the study, subjects that have shown allergies to cosmetic products.

### 3.3 Test with capsaicin

Aiming to detect stinging or burning effects that usually appear in sensitive subjects within few min, prior to each application of the study product/vehicle, 10% of an aqueous solution of capsaicin was applied to all subjects, to the skin rich in sensory innervation of the groove extending between the base of the nose and the upper lip. After that, the subjects applied the vehicle treatment on one hemiface, and the corresponding active treatment on the other hemiface.

### 3.4 Composition of the tested products

*Product A*: Aquammunist® (Solía TSW from Cantabria, Spain + diatom algae extract) in an inert vehicle. *Product B*: Aquammunist® + *Annona cherimola* Fruit Extract, in an inert vehicle. *Product C*: Aquammunist® plus Calming Complex (*Annona cherimola* Fruit Extract + Niacinamide), in an inert vehicle.

### 3.5 Study protocol

Products A, B, and C were applied on the same panel (8 subjects each session) with a cross‐over approach, following an interval of 3 days of wash‐up period between each product application (Table 2).

**TABLE 2:** Products A, B, and C were applied in comparison to a control skin area treated with a vehicle formulation.

	Session 1	Session 2	Session 3
8 subjects	A	B	C
8 subjects	B	C	A
8 subjects	C	A	B

The tested product was applied by the investigator, by means of a soaked cotton swab, on right/left groove running between the base of the nose and the upper lip according to a previously defined randomization list. The contralateral side was treated with the vehicle formulation acting as negative control.

### 3.6 Evaluation methods

Stinging and burning sensation: The clinical score and the VAS score of the stinging/burning sensation were monitored at all checkpoints by the investigator and with the collaboration of the subject. The intensity of the perceived feeling of discomfort was recorded according to the clinical scores: 1 (no reaction); 2 (mild reaction); 3 (moderate reaction) and 4 (severe reaction). The intensity of the perceived discomfort sensation according to the VAS score: from 0: no reaction, to 10: severe reaction.

Digital pictures: At T0, Tmax, T5′ and T10’ digital pictures of the treated area were taken using Visia®‐CR (Canfield Scientific Europe, BV, Proostwetering, Netherlands). The instrument ensures a reproducible subject positioning between each checkpoint, and allows to take pictures with RBX® red filter (RBXr) technology, to enhance visualization of the skin features to analyze.

### 3.7 Evaluation times

T0: Application of a 10% capsaicin aqueous solution to the skin of both nasolabial fold sides, to induce the stinging/burning sensation; digital pictures were taken.

*T*_max_: When the discomfort peaked its maximum sensation (approximately 2/3 min after the application of the 10% capsaicin aqueous solution) the investigator scored the intensity of the perceived discomfort and applied the tested product with a soaked cotton stick, on right/left nasolabial fold according to a previously defined randomization list. The contralateral side was treated with the vehicle formulation acting as negative control; digital pictures were taken.

The investigator scored the intensity of the perceived discomfort: T1st: immediately after the first products application; T1’: the investigator scored the intensity of the perceived discomfort 1 min after products application; T2’: the investigator scored the intensity of the perceived discomfort 2 min after products application; T3’: the investigator scored the intensity of the perceived discomfort 3 min after products application.; T4’: The investigator scored the intensity of the perceived discomfort 4 min after products application; T5’: the investigator scored the intensity of the perceived discomfort 5 min after products application; digital pictures were taken; T10’: the investigator scored the intensity of the perceived discomfort 10 min after products application; digital pictures were taken.

### 3.8 Statistical analysis

Data were submitted to Wilcoxon signed‐rank test. The intra‐group statistical analysis was carried out versus *T*_max_. The inter‐group statistical analysis was carried out on treated versus control area data, at each experimental checkpoint. Moreover, data are submitted to Friedmann's test for the inter‐group analysis. Variations are considered statistically significant when the *p* ≤ 0.05. The statistical software used for statistical analysis is NCSS 10 (version 10.0.7 for Windows; NCSS, Kaysville, UT, USA).

## 4 RESULTS

Twenty‐four (*n* = 24) subjects were enrolled. The mean age was 49 years old. This experimental setting was designed to assess the calming effect of the 3 tested products on the capsaicin‐induced stinging and burning sensation. (Table 2).

### 4.1 Clinical scoring of the stinging/burning sensation

#### 4.1.1 Product A versus vehicle

**FIGURE 1:**
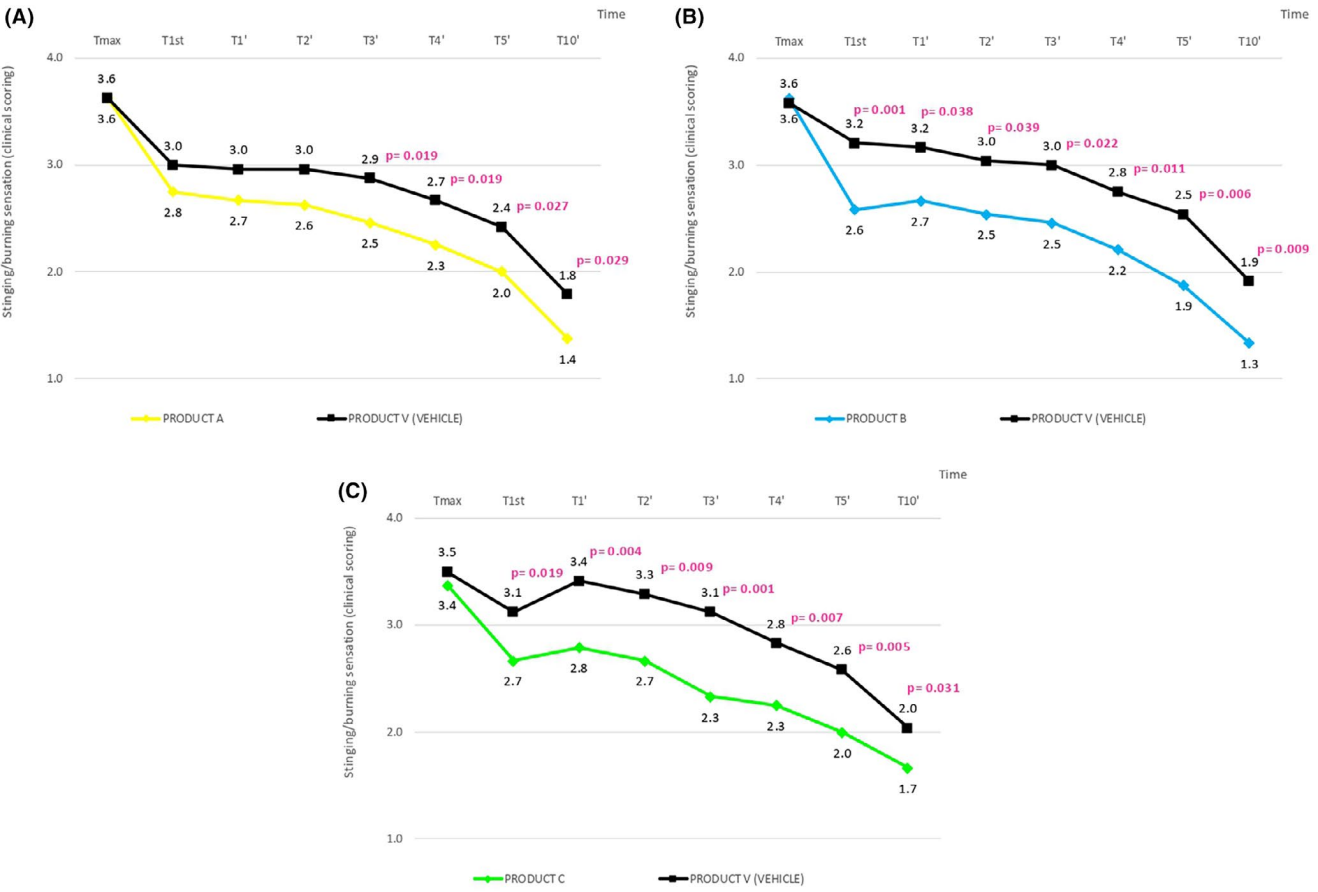
(A) Significantly higher decrease in discomfort sensations with product A since T3’. (B) Significantly higher decrease in discomfort sensations with product B since T1st. (C) Significantly higher decrease in discomfort sensations with product C since T1st. All graphs report the intergroup statistical analysis (vs. vehicle).

The single application of PRODUCT A determined a statistically significant decrease in the stinging/burning sensation induced by the capsaicin solution, starting from T1st (immediately after product application) and at each monitored checkpoint. This decrease was also recorded starting from T1st, in the vehicle‐treated skin. However, the inter‐group statistical analysis highlighted that the decrease of the discomfort sensation was higher after PRODUCT A application (*p* < 0.05) starting from T3'vs vehicle (Figure 1A).

#### 4.1.2 Product B versus vehicle

The single application of PRODUCT B determined a statistically significant decrease in the stinging/burning sensation induced by the capsaicin solution, starting from T1st (immediately after product application) and at each monitored checkpoint. This decrease was also significant within the vehicle‐treated side starting from T1st. However, the inter‐group statistical analysis highlighted that the decrease of the discomfort sensations was higher after PRODUCT B application (*p* < 0.05) starting from T1st (immediately after product application) versus vehicle. (Figure 1B).

#### 4.1.3 Product C versus vehicle

The single application of PRODUCT C determined a statistically significant decrease of the stinging/burning sensation induced by the capsaicin solution, starting from T1st (immediately after product application) and at each following monitored time. A significant decrease in the stinging/burning sensation was also recorded starting from T3’ in the vehicle‐treated skin, showing a faster decrease in discomfort sensations within the Product C treated side. In addition, the inter‐group statistical analysis highlighted a significantly higher decrease of the discomfort sensations with PRODUCT C (*p* < 0.05) vs. vehicle starting from T1st indicating a better soothing effect of PRODUCT C versus vehicle (Figure 1C).

### 4.2 VAS score of the stinging/burning sensation

The data obtained with VAS scale were in accordance with the previous clinical scoring.

PRODUCT A, B, C, and vehicle determined a statistically significant decrease of the stinging/burning sensation induced by the capsaicin solution (VAS score), starting from T1st and at each monitored checkpoint.

**FIGURE 2:**
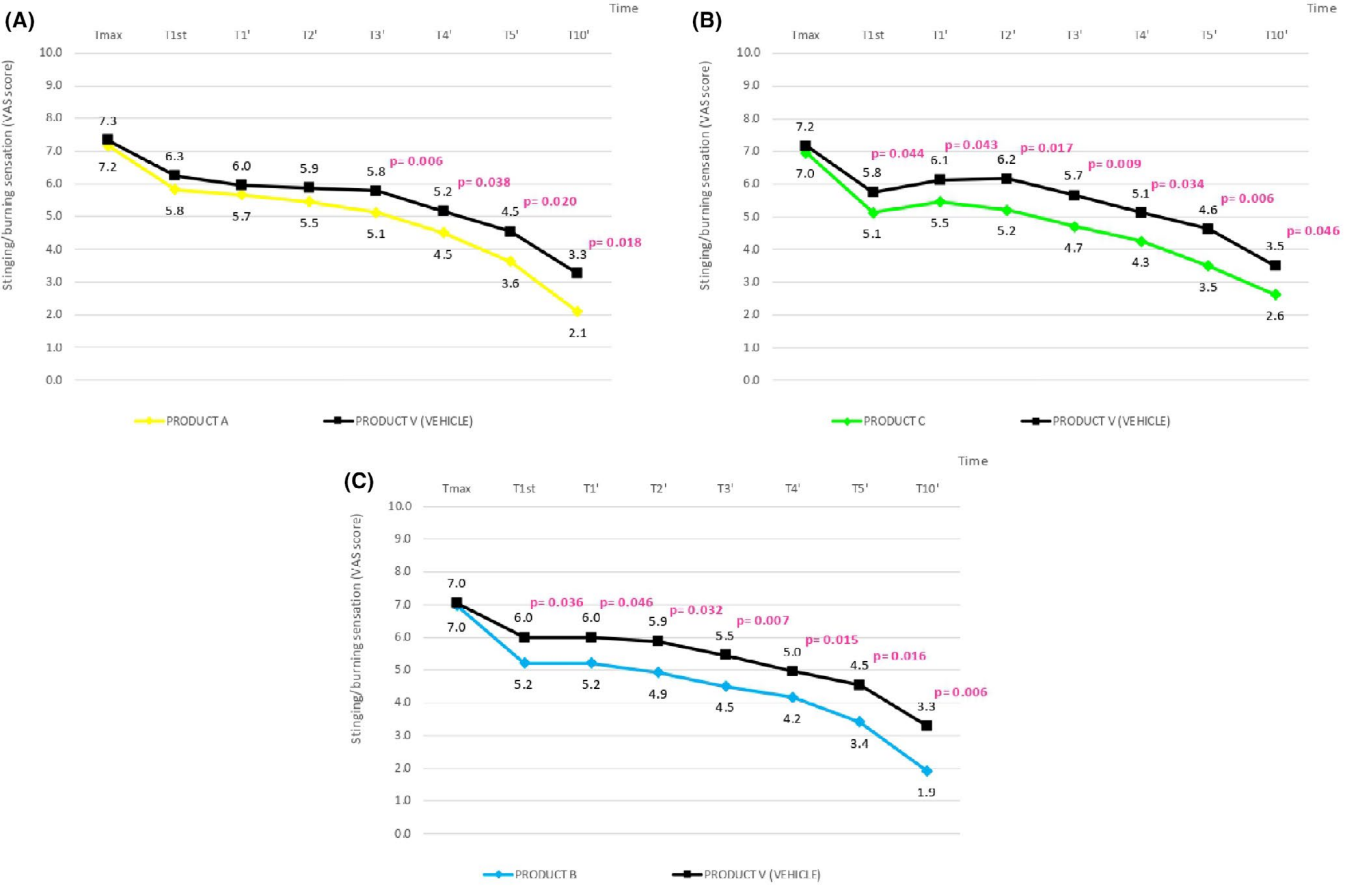
Significantly higher decrease in discomfort sensations with product A, B, and C versus vehicle. The graph reports the intergroup statistical analysis (vs. vehicle).

The inter‐group statistical analysis (active product vs. vehicle) showed that from T3’ the decrease of the discomfort sensations was significantly higher (*p* < 0.05) with the application of product A at T3’, and from T1st with the application of product B and C, indicating a better soothing effect of the three active products versus vehicle (Figure 2).

To note, no statistically significant difference was observed between PRODUCT A, PRODUCT B and PRODUCT C. The reaction to capsaicin induced in all patients a sensation of discomfort (stinging/burning sensation) with an overall degree of severity from moderate to severe, between 2 and 3 min after application.

All products tested, including the vehicle, improved capsaicin‐induced discomfort symptoms significantly since T1st, with the exception of the side treated with vehicle in the group comparing to Product C (Figure 2C), which started at T3’. No significant differences regarding speed of action were found between the groups. However, in all cases, the product tested demonstrated a better calming effect than the vehicle (statistically significant, *p* < 0.05). It should be noted that in T1, when product C is studied, a worsening was observed on the side treated with vehicle, while on the side treated with product C a significant improvement was already observed from T1st.

**FIGURE 3:**
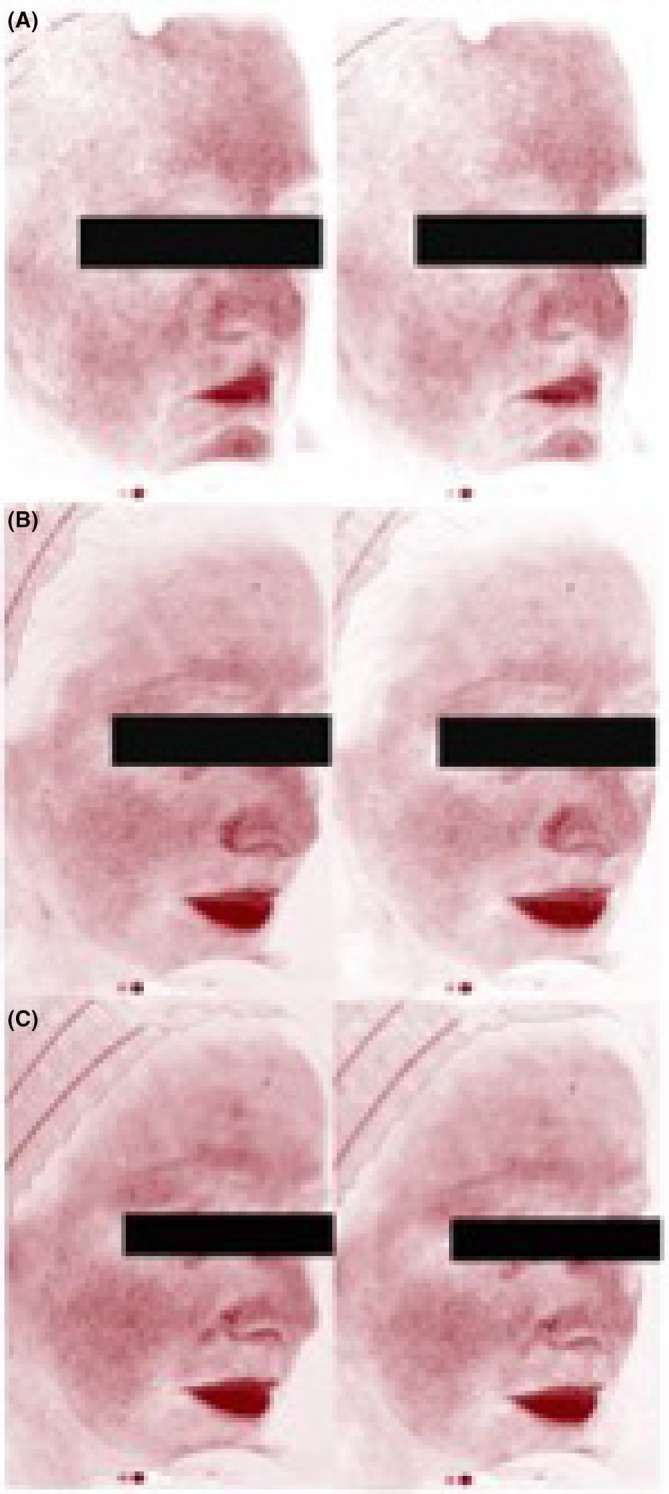
(A) Product A, (B) Product B and (C) Product C images at Tmax (left) and T5’ (right) respectively, showing the decrease in vascularization.

The images taken with Visia‐CR RBX® red filter showed a decrease in redness and vascularization (Figure 3).

## 5 DISCUSSION

There is no simple treatment routine for managing the SS. The avoidance of possible triggers and the use of well‐tolerated cosmetics, especially those that help control unpleasant sensations, are general recommendations for patients with SS. Photoprotection should also be encouraged in all patients.^36^ SS patients should choose products containing few or no preservatives and surfactants.^22^ In addition, actives with moisturizing properties would be helpful, as SS patients have higher moisturizing needs. Ferreira et al, in his review article about SS treatment, concluded that clinical studies designed to evaluate the benefits of cosmetic products in volunteers with SS are lacking.^35^ Therefore, we have done a clinical trial testing different ingredients that are supposed to improve symptoms of SS, as well as their combination.

Niacinamide is one of the most frequently used active ingredients in cosmetic products for SS according to a review article.^35^ This is why the inclusion of niacinamide in formulations for these types of patients is supported. Likewise, ingredients of marine origin and thermal waters have been explored because they have calming and anti‐inflammatory potential,^27^ supporting the inclusion of Aquammunist® in the study. In addition, as mentioned in the introduction, Annona plant extracts are able to stimulate the skin's endogenous adaptive mechanisms controlling the hyperactivation of TRPV1 receptors.^31^, ^32^, ^33^ As previously discussed, in SS there is a higher stimulation of TRPV1 leading to excessive cytokine secretion that triggers the neurogenic inflammatory cascade (which is associated with redness, itching, etc.) and alterations in barrier function.^21^, ^22^ That is the rationale why the use of cosmetics with effects on cutaneous nervous inflammation, especially controlling TRPV1 stimulation, should be encouraged in SS.^37^, ^38^ Thus, in this trial and following an innovative approach, the contribution of *A. cherimola* Fruit Extract as a new ingredient not directly blocking TRPV1, but modulating its action through adaptive mechanisms, is tested. For everything considered, each of the ingredients tested are theoretically interesting to be included in SS formulations.

For the treatment of SS, synergies of different assets are sought. In the products studied, different active ingredients with associated biological properties were included. On one hand, Aquammunist® is already a combination of two independent ingredients Solía TSW and diatom algae (*P. tricornutum*) extract. Based on literature, this combination would provide anti‐inflammatory and barrier function repair activities,^29^, ^30^ controlling the secretion of different proinflammatory cytokines.^29^ In fact, the benefits of this specific combination have already been reported in vitro with favorable results (manuscript submmited). On the other hand, the combination of Aquammunist® with *A. cherimola* Fruit Extract (which reverts the hyperactivation of TRPV1 receptors^31^, ^32^, ^33^ secondarily contributing to decreasing the induction of inflammatory cytokines^34^) was also tested as “treatment B”. Finally, treatment C which adds the effect of niacinamide, providing anti‐inflammatory action and barrier function protection, was also tested. Accounting for the results presented here, the improvement seen in these patients seems to support the combination of the mentioned ingredients.

The results showed a clear benefit in soothing effect associated with the products tested. Even when a certain calming effect induced by the vehicle was found, that could be explained by its water and pentylene glycol content, the differences were statistically significant in favor of the actives, showing a significantly greater decrease in unpleasant sensations with the different products evaluated vs vehicle. Therefore, these ingredients combination seem to contribute repairing the altered barrier, increasing the skin's resistance threshold. This relief was immediate and was also reflected in the reduction of the vasodilation seen in the photographs. For daily practice, this would suggest that it would be helpful to recommend the use of these actives to calm the discomfort in SS patients after a trigger, to induce instant relief by soothing the impaired skin barrier of the irritated skin. Taking into account that once the skin is irritated, its barrier also worsens and this facilitates further irritation, we would propose the use of these active ingredients in the daily care of people with SS, since by promoting an increase in the response threshold and improving the barrier function, they could improve the syndrome as such, avoiding episodes of irritation.

Limitations of our study would be the reduced number of participants; and that redness and vascularization were prospectively addressed by imaging when the data analysis could benefit from the implementation of a quantitative method.

In the future, it would be interesting to study the effect of daily routines that include this type of active ingredients.

## 6 CONCLUSIONS

All the three tested cosmetic combinations showed to act significantly better in calming the symptoms induced by capsaicin when compared to the vehicle, as the decrease in the severity of symptoms was greater after the application of the tested products compared to vehicle.

Therefore, with a great safety profile, the three cosmetic combinations formulated with ingredients of natural origin would be a valid option for patients with SS who are looking for suitable and effective products for their skin.

## AUTHOR CONTRIBUTIONS

All authors have read and agreed to the published version of the manuscript.

## FUNDING INFORMATION

This research was funded by Industrial Farmacéutica Cantabria (NIF A39000914), S.A., Cantabria, Spain.

## CONFLICT OF INTEREST STATEMENT

The authors declare that they work in Medical Affairs and in the R&D Department of Cantabrialabs. The funders participated in the design of the study, as well as in the analyses and interpretation of data.

## 7 ETHICS STATEMENT

The study was conduted following all ethical standards. The study was conducted in accordance with the Declaration of Helsinki. Ethical review and approval were waived for this study. According to the EU cosmetic Regulation no. 1223/2009, the cosmetic product must not cause damage to human health when applied under normal or reasonably foreseeable conditions of use and must be assessed for its safety of use before human subjects are exposed to it, and as such, further ethical approval is not required.

## CONSENT

Informed consent was obtained from all subjects involved in the study.

## Data Availability

The data that support the findings of this study are available from the corresponding author upon reasonable request.
